# Novel Dual-Component Microencapsulated Hydrophobic Amine and Microencapsulated Isocyanate Used for Self-Healing Anti-Corrosion Coating

**DOI:** 10.3390/polym10030319

**Published:** 2018-03-14

**Authors:** Maolian Guo, Wei Li, Na Han, Jianping Wang, Junfeng Su, Jianjie Li, Xingxiang Zhang

**Affiliations:** 1 State Key Laboratory of Separation Membranes and Membrane Processes, Tianjin Key Laboratory of Advanced Fibers and Energy Storage, School of Material Science and Engineering, Tianjin Polytechnic University, Tianjin 300387, China; guomaoliansx@163.com (M.G.); hannapolyu@163.com (N.H.); jpwangcn@gmail.com (J.W.); sujunfeng@tjpu.edu.cn (J.S.); zhangpolyu@aliyun.com (X.Z.); 2 Tianjin Colouroad Coatings & Chemicals Co., Ltd., Tianjin 300457, China; 18222858076@163.com

**Keywords:** self-healing, Microcapsule, isophorone diisocyanate, polyaspartic acid ester, in situ polymerization

## Abstract

Dual component microencapsulated hydrophobic amine and microencapsulated isocyanate were designed and fabricated for self-healing anti-corrosion coating. In this system, novel hydrophobic polyaspartic acid ester (PAE) and isophorone diisocyanate (IPDI) were microencapsulated respectively with melamine-formaldehyde (MF) as shell via in situ polymerization. To reduce the reaction activity between shell-forming MF prepolymer and PAE, another self-healing agent tung oil (TO) was dissolved in PAE and subsequently employed as core material. With field-emission scanning electron microscopy (FE-SEM) and optical microscopy (OM), the resultant microencapsulated IPDI with diameter of 2–5 μm showed a spherical shape and smooth surface. More importantly, both the morphology and microstructure of microencapsulated PAE enhanced significantly after addition of TO. Fourier transform infrared spectra (FTIR) analysis confirmed the molecular structure of chemical structure of the microcapsules. Thermal gravimetric analysis (TGA) indicated that both kinds of microcapsules exhibit excellent thermal resistance with the protection of MF shell. Furthermore, the self-healing epoxy coating system containing microencapsulated IPDI and microencapsulated PAE/TO was prepared and investigated. From the micrographs of true color confocal microscope (TCCM), the self-healing coating containing dual-component microcapsules showed excellent self-repairing performance compared to single microencapsulated IPDI system, and the optimal content of dual-component microcapsules in epoxy coating was 20 wt % approximately.

## 1. Introduction

Corrosion of metals has become a worldwide issue, which may lead to severe structure failure and dramatic economic loss [1]. One of the easiest and most common ways to protect metallic material against corrosion was the application of organic coatings. Such a coating layer could form a physical barrier to prevent contact between the metal surface and the corrosive environment (e.g., air, oxygen, and water). However, organic coatings were always subjected to mechanical damages such as scratches or cuts, which might cause failure of coatings, and water would penetrate through the coating then resulted in corrosion of metal [2,3]. Therefore, the concept of self-healing coating has been proposed to act as a quick remedy for mechanical damages. Self-healing referred to the material that could imitate the life system, had dual functions of perception and stimulation, and once the material had defects, it could self-repair without any outside action [4,5].

Since the first generation of self-healing material based on the ring opening metathesis polymerization (ROMP) between encapsulated dicyclopentadiene (DCPD) and Grubbs’ catalyst particles was researched [6], microencapsulation has been considered as one of the most efficient and popular approaches in self-healing development. The self-healing of microencapsulation [2,5] meant that when damage occurred in coating, the embedded microcapsules were able to crack and release repairing active chemicals to recover the defect. Encapsulation of healing agents could be carried out by various methods including in situ polymerization, interfacial polymerization, internal phase separation and solvent evaporation technique. Various shell materials could be synthesized through above mentioned techniques, such as urea-formaldehyde resin (UF), melamine-formaldehyde (MF), polyurea (PU), poly methylmethacrylate (PMMA) and silica [7,8]. Meanwhile, DCPD, poly dimethylsiloxane (PDMS), glycidylmethacrylate, epoxy, isocyanates, drying oil, and aliphatic amines and so on are selected as typical healing agents and microencapsulated [8,9].

Isocyanates, such as isophorone diisocyanate(IPDI) [10,11,12], hexamethylene diisocyanate (HDI) [13,14,15], HDI trimer [16] and polyaryl polymethylene isocyanates (PAPI) [17] can react with moisture and are being used as potential healing agents to develop catalyst-free self-healing materials in moist or aqueous environments. An extensive research on microencapsulation of isocyanate as repair agent and shell functionalization have been carried out in recent years. Yang et al. made the first attempt to encapsulate liquid IPDI via interfacial polymerization [18]. However, toxic toluene 2,4-diisocyanate (TDI) or 4,4-diphenylmethane diisocyanate (MDI) were often inevitably employed to form the PU shell during the interfacial polymerization. Besides, it appears that rapid microcracks propagation always occurred in the organic coating, while the reaction speed between isocyanate and water is usually slow especially in a low-humidity environment and low temperature [11,18]. Also, there listed the healing temperature, time and efficiency about isocyanate and some other healing agents in Table 1.

A thiol-isocyanate healing system was proposed by Xander et al. [22] to enhance the repair speed, and they added tertiary amine to accelerate the reaction speed between HDI and isooctyl 3-mercaptopropionate. The isocyanate conversion could be completed within several minutes, while little reaction could be found without the amine catalyst even after 1 h. Moreover, as a strong nucleophile, amine could react with isocyanate directly at room temperature, so the isocyanate-amine system showed great potential. However, it was proved difficult to encapsulate the mentioned amines due to their high reactivity and hydrophilicity [23]. Huan et al. reported a tetraethylenepentamine (TEPA) microcapsule with pickering emulsion templates [24], in which toxic solvents such as xylene and acetone were employed, however, it is difficult to separate and obtain the fabricated microcapsules from water-in-oil (W/O) system.

In this work, a novel isocyanate-amine system with fast healing speed was investigated to tackle the disadvantages mentioned above. We selected a new hydrophobic amine PAE [25,26] instead of traditional water-soluble amine, so both of hydrophobic IPDI and PAE could form O/W emulsion and be microencapsulated with melamine-formaldehyde (MF) resin as shell through in situ polymerization subsequently. During the synthesis of amine microcapsules, the residual hydroxyl of shell-forming prepolymer could also react with amino groups of PAE. In order to reduce the consumption of amine and facilitate the microencapsulation, solvent tung oil (TO) was added in PAE and composed as the core material of amine microcapsules. Furthermore, as a drying oil, the TO could also be used as another self-healing material, and they could polymerize through an autocatalytic oxidation reaction and form a hard, waterproof coating [27] when exposed to air for sufficient time. After IPDI and PAE/TO were microencapsulated by MF shell, respectively, the dual-component microcapsules dried powder was incorporated into epoxy resin solution and the self-healing performance was further studied systematically in this work.

## 2. Experimental

### 2.1. Materials

The melamine, paraformaldehyde and triethanol amine purchased from Tianjin Guangfu Fine Chemical Research Institute (Tianjin, China), were used to synthesize shell-forming MF prepolymer as shell. An aqueous solution of the sodium salt of styrene-maleic anhydride copolymer solution (SMA, 19 wt %) was kindly supplied by institute of functional fiber (Tianjin Polytechnic University, Tianjin, China) and employed as dispersant. As core materials, IPDI was purchased from Aladdin Industrial Corporation (Shanghai, China), PAE (solid content 97%) was purchased from Zhuhai Feiyang New Materials Corporation and (Zhuhai, China), TO was purchased from Jinan Haobang Chemical Corporation (Jinan, China) Epoxy resin (REF170, Shenzhen Hui Te Chemical Co., Ltd., Shenzhen, China) was employed as matrix material of self-healing coating. Sodium hydroxide and citric acid were both purchased from Fuchen Chemical Co., Ltd., Tianjin, China, which served as pH conditioning agent during microencapsulation. All chemicals were of reagent quality and used as received without further purification. 

### 2.2. Microcapsules Preparation

#### 2.2.1. Fabrication of MF Prepolymer

MF prepolymer was synthesized through the method reported by our previous research [28]. 7 g melamine and 4.8 g paraformaldehyde (molar ratio = 1/2.88) were mixed with deionized water in a 100 mL conical flask. The pH of suspension mentioned was set to 9 by adding triethanolamine. The system was stirred at 70 °C for 30 min until the solution became transparent, then the MF prepolymer solution was formed and remained on standby.

#### 2.2.2. Preparation of IPDI Microcapsules

IPDI microcapsules were synthesized via in situ polymerization in an O/W emulsion: 135 g deionized water, 15 g SMA and 20 g IPDI were mixed in a 400 mL beaker, then the mixture was emulsified by a homogenizer at 8000 rpm for 10 min to obtain a stable emulsion. 30 min of vacuum degassing (0.8 MPa) was used to remove the air bubbles and dissolved gas in the emulsion. Then the obtained emulsion was transferred into a three-necked round bottom flask and stirred under 400 rpm at 45 °C, then 15 g prepared MF prepolymer was dropped with speed of 0.75 g/min (the weight ratios of IPDI and MF was 3:1). The temperature of water bath was raised to 60 °C slowly, while 10 wt % citric acid solution was injected to regulate the pH to 5 approximately during the process of heating. After in situ polymerization continued for 2 h, the pH of system was adjusted to neutral to terminate polymerization, and then the microcapsule suspension was obtained.

#### 2.2.3. Microencapsulation of Amine

Amine microcapsules were synthesized via in situ polymerization in O/W emulsion as well. The specific synthesis process was according to method described above, and the rest synthetic parameters were also listed in Table 2 as below.

### 2.3. Preparation of Dual-Component Self-Healing Coating

The prepared IPDI microcapsules and amine microcapsules were intensively mixed with mass ratio of 1:2, then the dual-component microcapsules were filtered and washed 3 times with deionized water and dried at 60 °C for 12 h. Self-healing coatings were prepared by dispersing dual-component microcapsules (from 0 to 25 wt %) into epoxy resin solution. Metal plates with size of 10 cm × 10 cm × 0.2 cm were buffed off with sandpaper and degreased with ethanol, and then served as substrates. The prepared self-healing coatings were applied to the dried metal plates with thickness of 200 μm approximately. After solidified at room temperature for 24 h, all samples were scratched with a scalpel and placed in open air for 12 h. Micrographs were taken to compare the self-healing results of all samples.

### 2.4. Characterization

Optical microscope (OM, JNOEC, XSP-16A, Jiangnan Yongxin Company, Nanjin, China) equipped with computer was used to observe the emulsion droplets and microencapsulating process.

Field emission scanning electron microscope (FE-SEM, Gemini 500, Carl Zeiss, Germany) was used to observe the morphology, size, and shell thickness of obtained microcapsules. Samples were dispersed on conductive carbon adhesive tapes attached to aluminum stub, and then coated a layer of gold. The diameters of microcapsules were gauged using professional software (Nano Measurer 1.2.5, Fudan University, Shanghai, China) on the FE-SEM micrographs.

Fourier transform infrared spectroscopy (FTIR, NICOLET, IS10, Thermo Fisher Scientific, Shanghai, China) analyzer was used to characterize the chemical structures of the microcapsule sample, and the FTIR spectra of samples were obtained in the wave length range of 400–4000 cm^−1^.

The thermal stability of microcapsules was investigated using a thermogravimetric analyzer (TGA, STA449F3, Netzsch, Germany) at a scanning rate of 10 °C·min^−1^, from 20 to 1000 °C under nitrogen atmosphere. The thermal stability of microcapsules was characterized by measuring the weight (mass) loss with the temperature increase. 

The repair results of self-healing coating were observed by true color confocal microscope (TCCM, CSM700, Carl Zeiss, Germany), and the confocal mode was used to obtain 3D surface micrographs. The healing efficiency of coatings were gauged using professional software (ImageJ 1.48, National Institutes of Health, Bethesda, MD, USA ) on the micrographs.

## 3. Results and Discussion

### 3.1. Self-Healing Agent and Microencapsulation Mechanism

**Figure 1:**
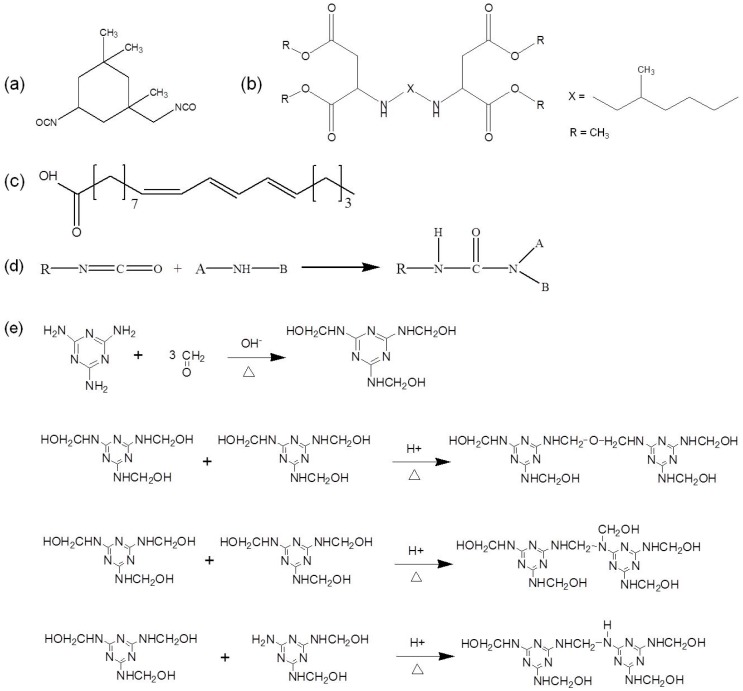
Chemical structure of (**a**) IPDI, (**b**) PAE and (**c**) alpha-eleostearic acid (primary component of TO); (**d**) reaction formula between –NH and –NCO, (**e**) polycondensation reaction of MF prepolymers.

To enhance the repair effect, dual-component microcapsules were selected and investigated in this work. The healing agents were chosen based on their adhesive capability, low toxicity, and thermal stability. As an aliphatic diisocyanate, IPDI shown in Figure 1a was selected as one part of repair agents with two active –NCO active groups. These inherent characteristic features such as high solids content, low viscosity and high reactivity of hydrophobic PAE, also made it potentially suitable for utilization in high-performance coatings and subsequent potential scale-up of production. The –NH groups located in the molecular structure (Figure 1b) could be sterically hindered aliphatic secondary diamine, while they could react with –NCO of IPDI at room temperature, and the reaction (as shown in Figure 1d) usually proceeded within several minutes. The resultant product polyurea presents many advantages such as flexibility, weather resistance, water resistance and wear resistance. As a drying oil obtained from the nut of the tung tree, TO was selected as both solvent of PAE and a secondary-phase healing agent. TO was a triglyceride mainly composed of alpha-eleostearic acid, and the chemical structure of which was presented in Figure 1c.

Owing to its low price, simple fabrication, good seal tightness and endurance, fire resistance, acid and alkaline resistance, MF resin was selected as the shell material. The formation of MF polymer was carried out by two steps as described in Figure 1e. The first stage was the process of methylolation to form MF prepolymer. The nucleophilic addition between melamine and formaldehyde would occur in weak alkaline medium, resulting in a mixture of different methylolated melamine, and the trimethylolmelamine is more stable by comparison. The second stage was the resinification, and polycondensation reaction was carried out in acidic medium to form bridges between the triazine rings. There may exist two mechanisms: dehydration condensation of two methylol groups to form the ether bridge, and condensation of methylol and amino groups to form the methylene bridges. Then further polymerization occurred to form an insoluble and non-melting polymer with a bulk structure finally.

**Figure 2:**
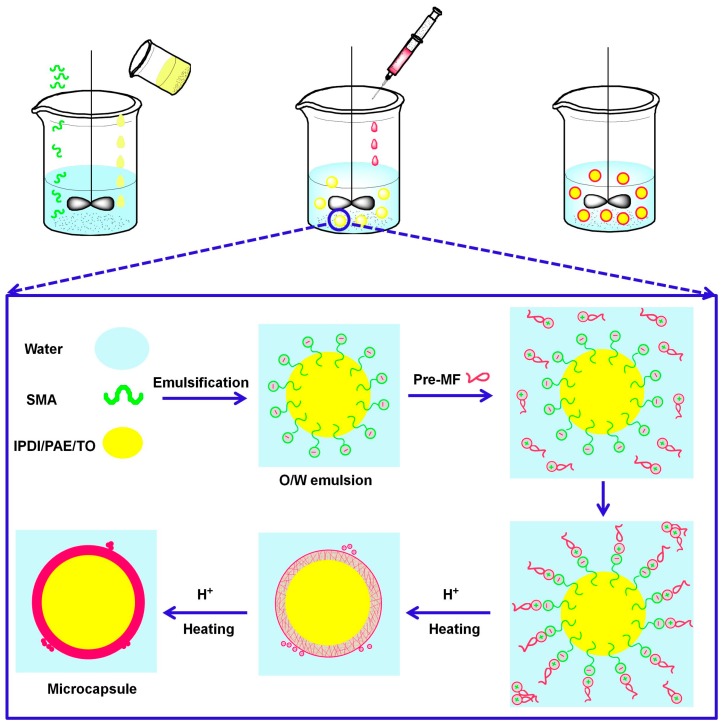
Schematic fabrication process of self-healing microcapsule by in situ polymerization.

Self-healing microcapsule with MF shell was prepared by in situ polymerization, and the schematic fabrication was shown in Figure 2. The hydrophobic phase (i.e., IPDI, PAE, PAE and TO) was added into the aqueous phase containing SMA and then emulsified to form a stable oil-in-water (O/W) emulsion. As an emulsifier, the molecular chain of SMA contains phenyl hydrophobic groups and the carboxyl hydrophilic groups, so they could be easily adsorbed to the interface during emulsification process and form stable emulsion. At the same time, the carboxylic ion groups located on SMA could absorb negative charge, while the amino groups on MF prepolymer could carry with positive charge [29]. Both kind groups could migrate to the oil-water interface with the electrostatic interaction after MF prepolymer was added, and then polymerization started to form the insoluble cross-linked shell at certain temperature and acidic environment. There also existed some nanoparticles on the shell surface of microcapsule, which might be resulted from self-polymerization of MF prepolymer when the electric attraction became weaker as the microencapsulating proceeded. 

### 3.2. Microencapsulation of IPDI

**Figure 3:**
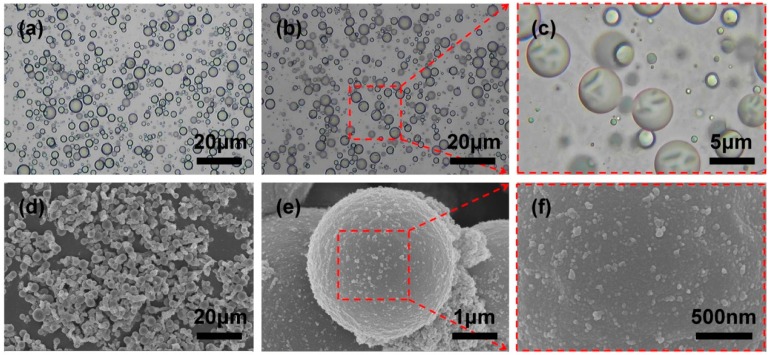
OM micrographs of (**a**) O/W emulsion, (**b**,**c**) the growth process of MF shell encapsulating IPDI core; (**d**–**f**) SEM micrographs of IPDI microcapsules with different magnification and micromorphology surface of microcapsule.

The OM micrographs in Figure 3 displayed the morphology evolution of the IPDI microcapsules. Firstly, The O/W emulsion with uniform particle size could be obtained facilely by high-speed shear emulsification as shown in Figure 3a, where obvious oil-water droplets interface could be observed clearly. After MF prepolymer as shell-forming monomer was added dropwise into the emulsion, the temperature was raised to 60 °C and maintained for 20 min, then the surface of the emulsion droplets became wrinkled as shown in Figure 3b,c, indicating that the MF prepolymer aggregated around the surface of emulsion droplets under charge attraction and began to polymerize to form the solid shell of microcapsules. After continuous polycondensation for 2 h under above mentioned reaction condition, IPDI microcapsules with MF-coated shell were finally obtained. The synthesized microcapsules were spherical shaped with average diameter of around 3.2 μm as Figure 3d,e shown, which was in accordance with the size of emulsion as well, so it confirmed the excellent stability of emulsion. Besides, there were some small solid particles with size less than about 100 nm on the surface, this could attribute to the fact that self-polymerization of prepolymer in water then adhered to the surface of microcapsules or gathered together in other existing mode. Moreover, the microcracks performance of microcapsules during self-healing process are greatly affected by the particle size. A partial enlargement of the capsule surface was shown in Figure 3f, the outer surface was found quite smooth and compact, thus contributing to encapsulate the core material without leakage. 

**Figure 4:**
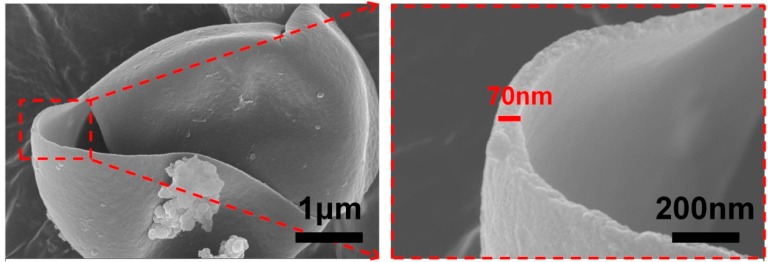
SEM micrographs of broken IPDI microcapsule.

The IPDI microcapsules were more likely to be ruptured when subjected to certain external force, the micrographs of a single broken microcapsule were shown in Figure 4. This illustrated that the shell material was sensitive to external forces, so the self-healing microcapsules distributed in substrate coatings could be ruptured when the substrate subjected external stimulate, and then released IPDI to target areas via capillary effects. In addition, we could find that the shell thickness of microcapsule was around 70 nm uniformly, and the inner surface of shell appeared smooth and compact. 

**Figure 5:**
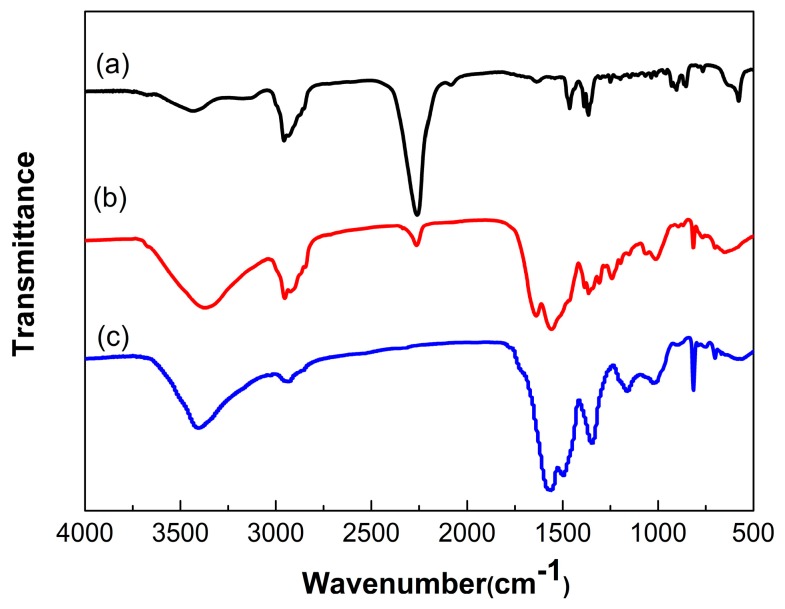
FTIR spectra of (**a**) IPDI; (**b**) microencapsulated IPDI and (**c**) MF shell.

To determine the successful microencapsulation, FTIR spectra was measured to identify the chemical structure of the microcapsules qualitatively, and the FTIR spectra of IPDI, microencapsulated IPDI and MF shell was shown in Figure 5. The absorption bands at 2260 cm^−1^ corresponding to –NCO stretch characteristic could be observed obviously on the spectrum of IPDI. The peaks at 2980 and 1470 cm^−1^ referred to the stretching and bending vibration of –CH, moreover, stretching and bending vibration peaks of –CH_2_ appeared at 2930 and 1380 cm^−1^. The MF shell material could be manifested with wide stretching vibration peaks of N–H at about 3353 cm^−1^ and stretching of triazine ring at 820 cm^−1^. The peak at 2930 cm^−1^ belonged to the stretching vibration of aliphatic –CH_2_, and the peaks of 1366–1556 cm^−1^ related to the stretching vibration of C=N. In addition, the stretching vibration peaks of C–N at 1000–1350 cm^−1^ also existed in spectrum of shell, the peak of 2260 cm^−1^ corresponding to –NCO and 3353 cm^−1^ belonging to N–H and triazine ring at 820 cm^−1^ appeared in the spectrum of microcapsules. The results indicated that IPDI was probably encapsulated with MF shell.

**Figure 6:**
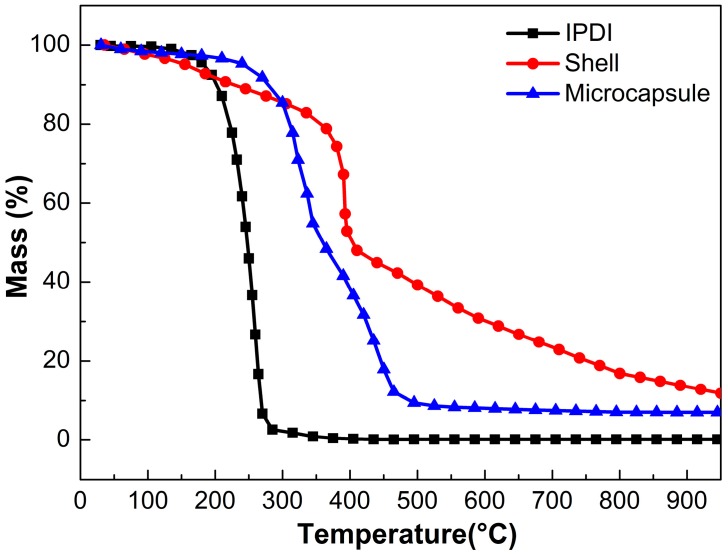
TGA curves of IPDI, microencapsulated IPDI and MF shell.

The thermal stability of self-healing microcapsule is a key parameter for its practical application, therefore, TGA measurements of IPDI, microencapsulated IPDI and MF shell were performed as shown in Figure 6. As can be seen, the vaporization temperature (defined as 5 wt % mass loss) of IPDI was 163 °C approximately, while it increased to 236 °C after being encapsulated, and thus this illustrated that MF shell provided a thermal resistance for IPDI core, which could protect IPDI from permeation firmly. The shell material lost about 5% of weight below 150 °C presumably from cleavage of ether bond and elimination of H_2_O and formaldehyde. There still around 15% residual mass remained at 800 °C mainly due to residual carbon. Besides, the weight loss of microcapsules was 56.2 wt % approximately from beginning to 350 °C and then lost weight with a higher rate until 500 °C. Compared with the weight loss curves of IPDI and shell, IPDI evaporated completely and shell lost weight about 15% at 350 °C, so it was reasonable to conclude that the weight loss was attributed to evaporation of the IPDI, residual water and formaldehyde from MF shell. Therefore, the core fraction of microcapsules was estimated as 48.5 wt % approximately.

### 3.3. Microencapsulation of Hydrophobic PAE-TO

**Figure 7:**
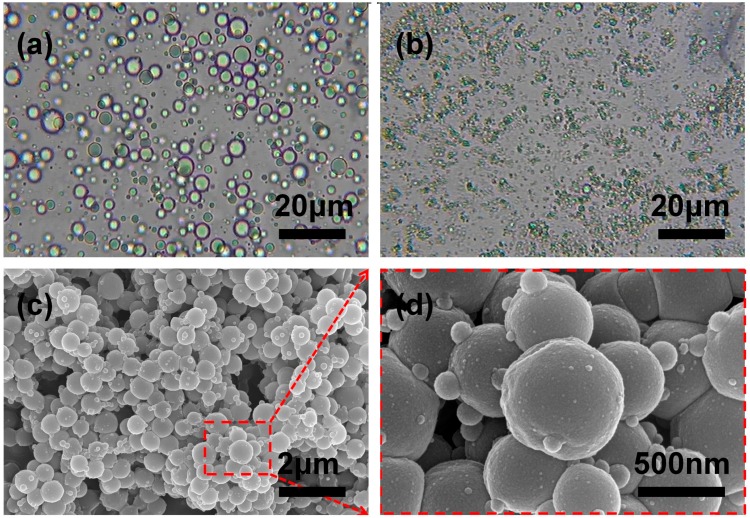
OM images of (**a**) O/W emulsion and (**b**) the growth process of MF shell encapsulating PAE core; SEM micrographs of (**c**,**d**) microencapsulated PAE with different magnification.

To facilitate microencapsulation, the hydrophobic amine PAE was selected as another component of self-healing system. As shown in Figure 7a, the stable O/W emulsion could be obtained after emulsification, and the size of droplets ranged from 2 to 5 μm. The emulsion was transferred to three-necked flask and MF prepolymer was dropped subsequently, however, it was worth to note that the particle size of oil droplets suddenly decreased from several microns to submicron or nanometer scale dramatically as Figure 7b shown. Furthermore, from the SEM image of Figure 7c, we could also find the PAE microcapsules exhibit smooth surface, and the mean diameter of microcapsules was 500 nm approximately, which was relatively uniform and consistent with the droplets’ size presented in Figure 7b. This phenomenon might be attributed to the fact that partial reaction between the amino groups of PAE and hydroxyl or of the MF prepolymer occurred, resulting in the obvious diameter decrease of PAE microcapsules. However, it is considered hard for the PAE capsule with submicron or nanometer scale diameter to be ruptured to release self-healing PAE, and also less active self-healing PAE remained inside the shells. 

**Figure 8:**
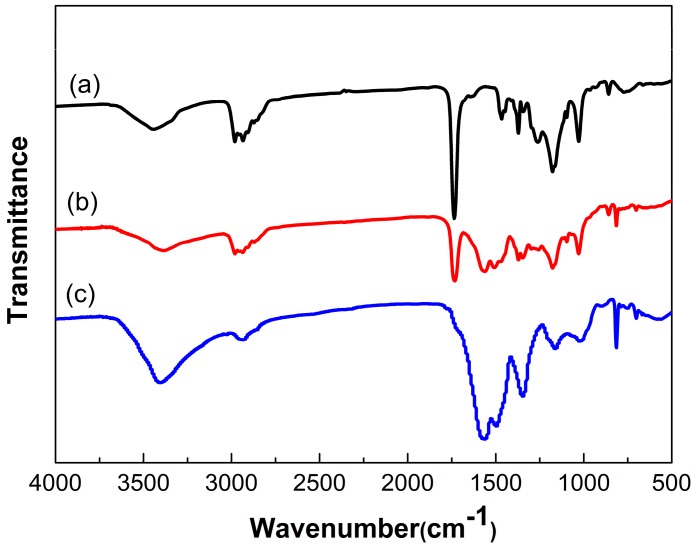
FTIR spectra of (**a**) PAE; (**b**) microencapsulated PAE and (**c**) MF shell.

As presented in Figure 8, the FTIR spectra of PAE, microencapsulated PAE and the MF shell were measured to further study the molecular structure of microcapsule. On the spectrum of PAE, besides the peaks of –CH and –CH_2_ related to stretching and bending vibration, the stretching and out-plane rocking vibration peaks of –NH appeared at 3340 and 858 cm^−1^ respectively, meanwhile, the stretching vibration peak of C–N appeared at 1028 cm^−1^, all of these characteristic peaks mentioned above were associated to amine group. The peaks at 1735 and 1177 cm^−1^ corresponded to the stretching and bending vibration of C=O in ester group respectively, and the peak at 1260 cm^−1^ related to the stretching vibration of C–O. On the spectrum of microcapsules, besides the characteristic peaks of shell appeared at about 3440 and 820 cm^−1^, all the peaks belonging to PAE appeared. These results indicated that PAE was probably microencapsulated with melamine formaldehyde resin shell.

**Figure 9:**
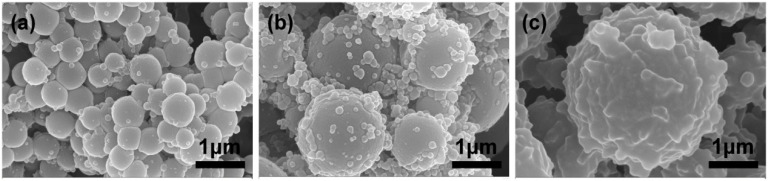
SEM micrographs of (**a**) microencapsulated PAE; (**b**) microencapsulated PAE/TO and (**c**) microencapsulated TO.

To solve the problem that core material partially reacted with the MF prepolymer, another self-healing agent TO was added also as solvent of PAE to reduce the reaction activity. TO and PAE are mutually soluble well, and TO could also employed as another self-healing material to consummate the dual-component repair system. We encapsulated PAE, PAE/TO (the mass ratio was 1:1) and TO under same encapsulation process, respectively. As shown in Figure 9, the morphology of microcapsules containing different core material were observed using FE-SEM. The diameter of microencapsulated PAE was measured to be 500 nm approximately (Figure 9a), and the particle size of microencapsulated TO was measured about ten times larger than that of microencapsulated PAE (Figure 9c), besides, the surface of microencapsulated TO became much rougher than that of microencapsulated PAE which could enhance the microcapsule/coating interface bonding. While for the mixed core of PAE and TO, the diameter of microcapsules reached about 2 μm (Figure 9b). This result indicated that the addition of TO could alleviate this problem effectively, and the confirmation of structure and properties required to be demonstrated by further characterization or other measurements.

**Figure 10:**
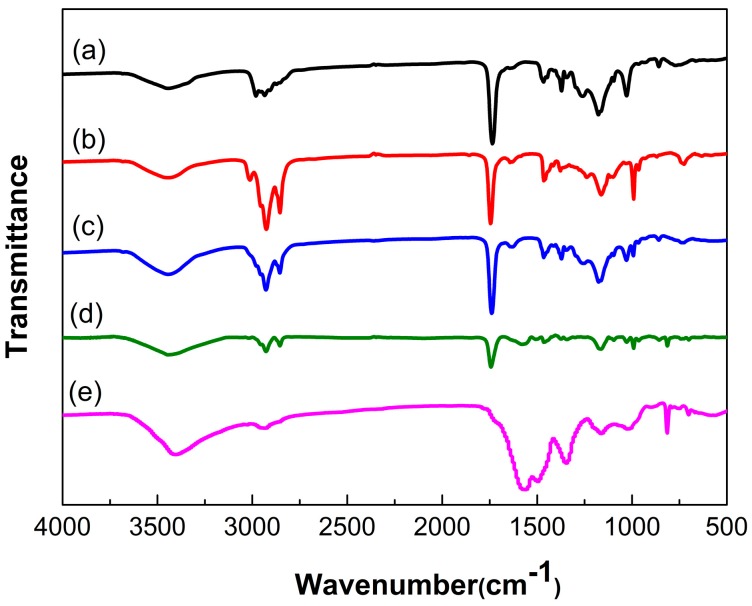
FTIR spectra of (**a**) PAE; (**b**) TO; (**c**) the mixture of PAE and TO; (**d**) microencapsulated PAE-TO and (**e**) MF shell.

Figure 10 showed the FTIR spectra of the PAE, TO, the mixture of PAE and TO, microencapsulated PAE/TO and the MF shell. The mixture spectra showed characteristic peaks of PAE at 3340 cm^−1^ (–NH stretching vibration), 858 cm^−1^(–NH out-plane rocking vibration) and 1028 cm^−1^ (C–N stretching vibration). The spectrum also presented the characteristic peaks of TO at 3012 cm^−1^ (=CH stretching vibration), 2927 and 2855 cm^−1^ (–CH stretching vibration), 1745 cm^−1^ (C=O stretching vibration) and 725 cm^−1^ (–(CH_2_)_n_− (*n* ≥ 4) bending vibration). FTIR curves confirmed that there was no new chemical bond generated during the mixing process of PAE and TO. In the spectrum of microcapsule, besides the characteristic peaks related to PAE and TO, the characteristic peaks of MF could be manifested at: 1366–1556 cm^−1^ corresponding to the stretching vibration of –C=N and 820 cm^−1^ associated to stretching of triazine ring. These results indicated that the mixture of PAE and TO as core was probably encapsulated by MF resin shell.

**Figure 11:**
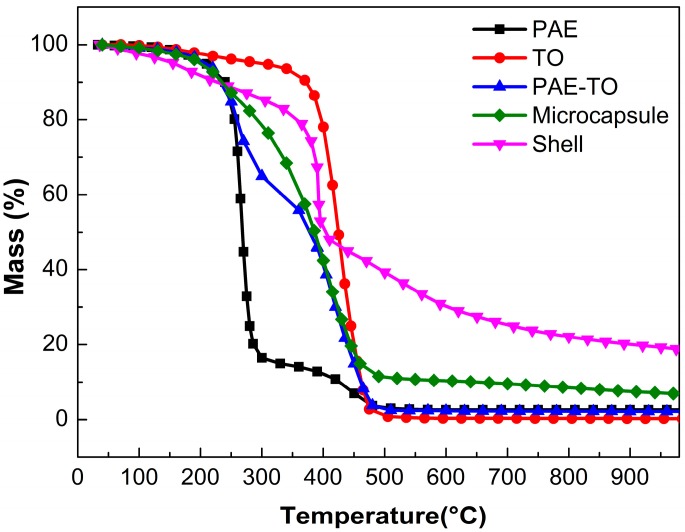
TGA curves of PAE, TO, the mixture of PAE and TO, microencapsulated PAE/TO and MF shell.

Thermogravimetry analysis were carried out for thermal degradation characterization of the amine microcapsules. Figure 11 showed the TGA curves of PAE, TO, the mixture of PAE and TO, microencapsulated PAE-TO and MF shell. Firstly, the PAE was thermally stable up to 252 °C and degradation occurred in two stages. The first stage at 252–285 °C lost weight about 85%, the second stage ranged from 416 to 500 °C and decomposed completely at 500 °C. TO started to decompose at about 330 °C as seen from its weight loss curve. On the curve of the mixture of PAE and TO there existed three stages, i.e., 233 °C corresponding to lose weight of about 35%, 365 °C corresponding to around 49.5% and 442 °C corresponding to 14%, and this further indicated that there were no chemical bond or new material formed during the mix process when the PAE/TO was 1:1. After being microencapsulated by MF resin, thermal decomposition of microcapsule started around 195 °C and continued up to 305 °C, this was mainly attributed to the cleavage of ether bond and elimination of formaldehyde from degradation of MF shell above 200 °C. The second stage of decomposition occurred between 305 and 502 °C, which was likely attributed to the evaporation or decomposition of mixture of PAE and TO encapsulated in the shell. The thermal degradations of the core and shell took place in overlapping temperature range (252–485 °C). 

### 3.4. Performance of Self-Healing Coating Containing Dual-Component Microcapsule

**Figure 12:**
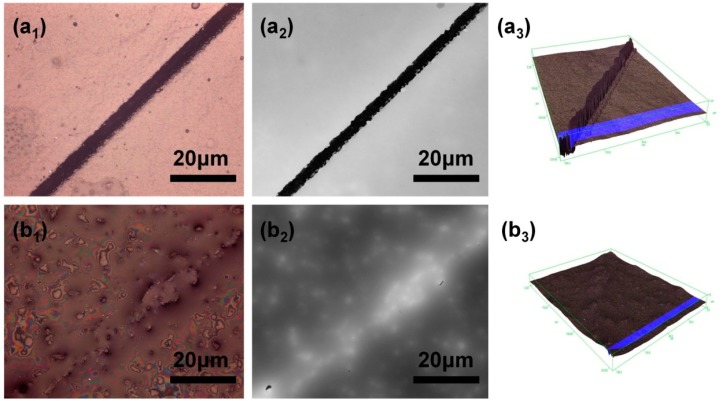
TCCM images of scratched regions (**a_1_**–**a_3_**) control coating without self-healing microcapsules; (**b_1_**–**b_3_**) self-healing coating containing self-healing microcapsules.

To evaluate the self-healing performance, the control coating and self-healing coating contained 25 wt % dual-component microcapsules were placed in the air for 12 h at room temperature (~20 °C). TCCM was used to observe the repair renderings, and the three-dimensional surface micrographs could also be obtained through confocal mode, besides, the roughness change of samples from 3D topography could be compared as well. As was clearly shown in Figure 12a_1_,a_2_, an obvious crack could be observed in the control coating sample. By contrast, it could be found that the scratched area of the substrate coated with self-healing coating was nearly fully free of crack as shown in Figure 12b_1_,b_2_. Furthermore, the repair results of crack could be found clearly by comparing the roughness of coating surface (Figure 12a_3_,b_3_). Repairing property of self-healing coating could mainly attribute to the incorporation of microencapsulated IPDI and microencapsulated PAE/TO. The released repair agents from ruptured microcapsules could heal the crack automatically by reacting with each other, and subsequently the newly-formed polyurea was able to fill microcrack. The result clearly demonstrated the great potential of self-healing coating containing dual-component microcapsule in external environments.

**Figure 13:**
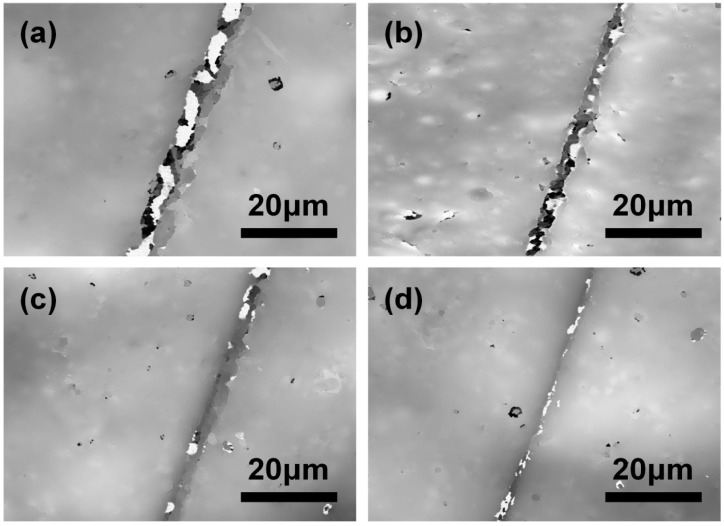
TCCM images of scratched regions with various contents of dual-component microcapsules: (**a**) 5 wt %; (**b**) 10 wt %; (**c**)15 wt % and (**d**) 20 wt %.

Here, dual-component self-healing coating containing various contents of microcapsules were prepared to investigate the self-repairing capability under air circumstance for 12 h at room temperature (~20 °C). As results shown in Figure 13, the crack was still obvious when 5 wt % self-healing microcapsules were added. With the content of microcapsules increased from 5 to 20 wt %, the repair of crack further tended to perfect. However, a small amount of micron-scale protrusions structure was formed on surface according to the images in Figure 12b_2_, which was mainly caused by excessive microcapsule accumulation, thus the properties such as smoothness, compactness and adhesion would be seriously affected when the content exceeds 20 wt %. Therefore, in comprehensive view, the appropriate content of microcapsules should no more than 20 wt % for optimal performance.

**Figure 14:**
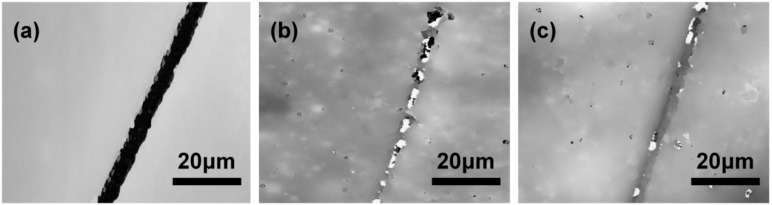
TCCM images of scratched regions with different kinds of microcapsules: (**a**) without microcapsule; (**b**) 15 wt % IPDI microcapsules and (**c**) 15 wt % dual-component microcapsules.

Furthermore, coatings containing different kinds of microcapsules were carried out to compare the self-healing effect of IPDI microcapsules and dual-component microcapsules in the air for 12 h at room temperature (~20 °C). From Figure 14a we found the crack was clearly visible in epoxy coating without any microcapsules. By contrast, the coating containing 15 wt % IPDI microcapsules was repaired partly as shown in Figure 14b, and this may be attributed to the slow reaction between IPDI and water vapor in the air, and the complete self-repair could not carry out in such a short time. In the end, the coating with dual-component microcapsules achieved optimal repair result presented in Figure 14c, which mainly resulted from that the fast reaction of isocyanate-amine nucleophilic addition and the newly-formed polyurea layer. The healing efficiency with different healing system and microcapsules content were compared and summarized in Table 3, the self-healing efficiency of dual-component microcapsules with content of 20 wt % could reached 98%. As a result, these results demonstrated the high efficiency of dual-component self-healing microcapsules and their potential application in anti-corrosion field.

## 4. Conclusions

In this work, hydrophobic amine polyaspartic acid ester (PAE) was combined with isophorone diisocyanate (IPDI) to provide dual-component self-healing system for epoxy matrix coating. The isocyanate-amine nucleophilic addition reaction was fast enough to ensure fast inhibition of crack propagation. Both PAE and IPDI were successfully microencapsulated with melamine-formaldehyde (MF) shell via in situ polymerization, respectively, the shell thickness of which was measured to be around 70 nm uniformly. Tung oil (TO) was added in PAE core both as self-healing regent and inhibitor to reduce the reaction activity between PAE and MF prepolymer during microencapsulating process. The characterization of OM, FE-SEM, FTIR and TGA demonstrated that both IPDI and amine microcapsules with core-shell structure were synthesized successfully. The microencapsulated IPDI and microencapsulated PAE/TO with diameter of 2–5 μm exhibited excellent thermal stability by elevating the temperature to 236 and 195 °C, respectively. The self-polymerized nanoparticles located on the shell surface increased their surface roughness and enhance the microcapsule/coating interface bonding accordingly, and the shell was sensitive to external stress. Furthermore, the self-healing system was prepared by incorporating microencapsulated IPDI and microencapsulated PAE/TO into epoxy coating, and the self-repairing performance was characterized by TCCM. The dual-component self-healing coating showed excellent self-repairing performance compared to IPDI single microcapsules system, and the appropriate content of dual-component microcapsules in epoxy coating was around 20 wt %. The dual-component self-healing microcapsules with good automatic healing properties presented potential application in anti-corrosion coating field.

## Figures and Tables

**Table 1:** The self-healing properties of different healing agents.

Healing Agent	Temperature (°C)	Time	Healing Efficiency (%)
drying oil [19]	25	2–4 day	54
Epoxy [8]	45	2 day	49
Epoxy-amine [20]	25	2 day	91
Isocyanate [21]	25	1 day	100
Thiol-isocyanate [22]	25	8 h	61

**Table 2:** Synthetic parameters of microencapsulated amine with MF resin shell.

Samples	Core Material Composition	Weight (g)	Emulsification Speed (rpm)
S1	PAE	20 g	3000
S2	PAE	20 g	5000
S3	PAE/TO	10 g/10 g	5000
S4	TO	20 g	5000

**Table 3:** Healing efficiency with different healing system and microcapsules content.

Self-Healing System	Content	Healing Efficiency
dual-component microcapsules	5 wt %	63%
dual-component microcapsules	10 wt %	84%
dual-component microcapsules	15 wt %	93%
IPDI microcapsules	15 wt %	78%
dual-component microcapsules	20 wt %	98%
dual-component microcapsules	25 wt %	100%
